# Blood transfusion in cardiac surgery is a risk factor for increased hospital length of stay in adult patients

**DOI:** 10.1186/1749-8090-8-54

**Published:** 2013-03-26

**Authors:** Filomena RBG Galas, Juliano P Almeida, Julia T Fukushima, Eduardo A Osawa, Rosana E Nakamura, Carolina MPDC Silva, Elisângela Pinto Marinho de Almeida, Jose Otavio Costa Auler, Jean-Louis Vincent, Ludhmila A Hajjar

**Affiliations:** 1 Surgical Intensive Care Unit and Department of Anesthesiology, Heart Institute (InCor), Hospital das Clinicas da Faculdade de Medicina da Universidade de Sao Paulo, Sao Paulo, Brazil; 2 Department of Intensive Care, Erasme Hospital, Universita Libre de Bruxelles, Brussels, Belgium

**Keywords:** Transfusion, Cardiac surgery, Length of stay

## Abstract

**Background:**

Allogeneic red blood cell (RBC) transfusion has been proposed as a negative indicator of quality in cardiac surgery. Hospital length of stay (LOS) may be a surrogate of poor outcome in transfused patients.

**Methods:**

Data from 502 patients included in Transfusion Requirements After Cardiac Surgery (TRACS) study were analyzed to assess the relationship between RBC transfusion and hospital LOS in patients undergoing cardiac surgery and enrolled in the TRACS study.

**Results:**

According to the status of RBC transfusion, patients were categorized into the following three groups: 1) 199 patients (40%) who did not receive RBC, 2) 241 patients (48%) who received 3 RBC units or fewer (low transfusion requirement group), and 3) 62 patients (12%) who received more than 3 RBC units (high transfusion requirement group). In a multivariable Cox proportional hazards model, the following factors were predictive of a prolonged hospital length of stay: age higher than 65 years, EuroSCORE, valvular surgery, combined procedure, LVEF lower than 40% and RBC transfusion of > 3 units.

**Conclusion:**

RBC transfusion is an independent risk factor for increased LOS in patients undergoing cardiac surgery. This finding highlights the adequacy of a restrictive transfusion therapy in patients undergoing cardiac surgery.

**Trial registration:**

Clinicaltrials.gov identifier: http://NCT01021631.

## Background

The rationale for perioperative red blood cell (RBC) transfusion is based on the observations that anemia is an independent risk factor for morbidity and mortality after cardiac surgery and that red blood cell transfusion would benefit a subset of patients presenting tissue hypoperfusion [1,2]. However, transfusions have been associated with high rates of morbidity and mortality in critically ill patients, and there is increasing evidence for independent relationships between RBC transfusion and infectious complications, cardiac and respiratory morbidity, prolonged length of stay (LOS) and mortality after cardiac surgery [2-4].

The Transfusion Requirements After Cardiac Surgery (TRACS) study was a prospective, randomized, controlled trial that recently demonstrated the safety of a restrictive strategy of transfusion compared with a liberal strategy in patients undergoing elective cardiac surgery [2]. In this study, independent of the adopted transfusion strategy, the number of transfused RBC units was an independent risk factor for clinical complications and death at 30 days [2].

The primary objective of this study was to assess the relationship between RBC transfusion and hospital length of stay in a large, single reference center of cardiac surgery. Secondary objectives were to compare the characteristics of patients who received RBC transfusion with those who did not, to evaluate the relationship of the number of transfused RBC units with mortality and clinical complications, and to identify the predictive factors for a prolonged hospital LOS. We hypothesized that patients requiring early postoperative blood transfusion would have longer hospital length of stay.

## Methods

### Study design

A detailed description of the TRACS study was reported previously [2]. The TRACS study was designed as a prospective, randomized, non-inferiority, controlled trial (Additional file 1). Consecutive patients who were scheduled for elective cardiac surgery with cardiopulmonary bypass between February 9, 2009 and February 1, 2010, were enrolled. We included patients who underwent elective coronary artery bypass graft (CABG) surgery or cardiac valve replacement or repair, alone or in combination (Additional file 1). The study was approved by the the Heart Institute Ethics Committee, Clinics Hospital, University of São Paulo, and written informed consent was obtained from all patients before enrollment. Patients were randomly assigned to a liberal or restrictive transfusion strategy. Patients from the liberal strategy group received RBC transfusions if their hematocrit values were lower than 30% from the beginning of the surgery until discharge from the ICU. Patients assigned to the restrictive strategy group received RBC transfusions if their hematocrit values were lower than 24%. At the Heart Institute, RBCs are separated from whole blood and stored in a citrate solution without leukodepletion. In this study, the median RBC unit storage time was 3 days.

In this substudy, we analyzed data from the overall population of 502 patients included in the TRACS study. Early postoperative RBC transfusion was defined as the total number of RBC units administered during the first 72 postoperative hours, including the operative room (Additional file 1). According to the number of units of RBC transfusion, patients were further categorized into the following three subgroups: 1) patients who did not receive RBC transfusion, 2) patients who received 3 RBC units or less (low transfusion group), and 3) patients who received more than 3 RBC units (high transfusion group). Hospital LOS was chosen as the primary outcome variable because it incorporates the cumulative effect of postoperative complications previously associated with blood transfusion.

Demographic data, intraoperative characteristics and clinical outcomes were obtained as previously described, and compared among groups [2].

### Statistical analysis

We compared baseline characteristics, intraoperative data and postoperative outcomes using Pearson’s chi-square test. These results are expressed as absolute numbers and as relative frequencies. The hospital length of stay is expressed as the median and 95% confidence intervals, assessed using a Kaplan Meier estimates curve. In addition, hospital length of stay is represented graphically as a box plot. The boxes indicate the first quartile, the median, and the third quartile. Open circles indicate the minor outliers (observation 1.5 × interquartile range [IQR] outside the central box); asterisks indicate the major outliers (observation 3.0 × IQR outside the central box). The association of preoperative and intraoperative factors with the need for RBC transfusion was assessed in a univariate logistic regression model. An independent association between the transfusion category and the primary outcome variable (LOS) was assessed using a Cox proportional hazards model. Each of the candidate variables was chosen from the univariate model for inclusion in the multivariate analysis Cox proportional hazards model. The variables that were independently associated with hospital LOS were included in the final multivariate analysis. All of the statistical tests were two-sided, and a *p*-value of less than 0.05 was considered statistically significant. These statistical analyses were performed using SPSS version 18.0 (SPSS Inc., Chicago, Illinois).

## Results

From the total of 502 patients randomized in the TRACS study, 199 did not receive any RBC transfusion (40%), 241 (48%) patients were given 1–3 units and 62 (12%) received more than 3 RBC units in the first 72 postoperative hours. Baseline characteristics of the groups are compared in Table 1. Female gender, age higher than 65 years-old, redo surgery, higher European System for Cardiac Operative Risk Evaluation (EuroSCORE) values and renal disease were associated with exposure to RBC transfusion. Other variables as preoperative left ventricular ejection fraction, heart failure, acute myocardial infarction, hypertension, diabetes, and unstable angina were not associated with exposure to RBC units.

**Table 1:** Baseline characteristics and intraoperative characteristics of patients according to the category of red blood cell transfusion

**Variable**	**Unit of RBC**			
	**None (N = 199)**	**1–3 units (N = 241)**	**>3 units (N = 62)**	**P**
Gender (Female)	49 (24.6%)	114 (47.3%)	29 (46.8%)	<0.001
Age (≥65 years)	57 (28.6%)	107 (44.4%)	30 (48.4%)	0.001
Redo surgery	3 (1.5%)	7 (2.9%)	14 (22.6%)	<0.001
EuroSCORE				
<3	126 (63.3%)	146 (60.6%)	35 (56.5%)	<0.001
3–5	63 (31.7%)	78 (32.4%)	18 (29%)	
>5	10 (5%)	17 (7.1%)	9 (14.5%)	
Left ventricular ejection fraction (%)				
<40	29 (14.6%)	31 (12.9%)	9 (14.5%)	0.738
40–59	53 (26.6%)	78 (32.4%)	20 (32.3%)	
≥60	117 (58.8%)	132 (54.8%)	33 (53.2%)	
Previous myocardial infarction	72 (36.2%)	80 (33.5%)	23 (37.7%)	0.755
Hypertension	159 (79.9%)	183 (75.9%)	51 (82.3%)	0.435
Diabetes	62 (31.2%)	75 (31.4%)	28 (45.9%)	0.075
Renal disease	15 (7.5%)	25 (10.7%)	12 (20.3%)	0.019
Unstable Angina	60 (30.2%)	74 (30.7%)	21 (33.9%)	0.855
Liberal RBC transfusion strategy	58 (29.1%)	147 (61%)	48 (77.4%)	<0.001
Procedure				
CABG	126 (63.3%)	146 (60.6%)	35 (56.5%)	0.164
Valve	63 (31.7%)	78 (32.4%)	18 (29%)	
CABG + valve	10 (5%)	17 (7.1%)	9 (14.5%)	
Cardiopulmonary bypass (>100 min)	59 (29.8%)	86 (35.8%)	27 (43.5%)	0.112
Initial hemoglobin (<13 g/dL)	64 (32.7%)	173 (72.7%)	51 (82.3%)	<0.001
Initial hematocrit (<39%)	45 (23%)	151 (63.4%)	47 (75.8%)	<0.001
Initial lactate (≥2 mmol/L)	55 (27.8%)	56 (23.5%)	10 (16.1%)	0.163
Initial SvcO_2_ (≥65%)	143 (87.2%)	163 (80.7%)	46 (83.6%)	0.248

Abbreviations: *RBC*, red blood cell transfusion;*EuroSCORE*, European System for Cardiac Operative Risk Evaluation; *Redo*, previous cardiac surgery, *CABG*, coronary artery bypass surgery; *SvcO*_*2*_, central venous oxygen saturation; *min*: minutes; *Initial* = samples collected after the induction of anesthesia.

Intraoperative characteristics for the RBC transfusion groups are compared in Table 1. As expected, a higher proportion of patients from the liberal strategy group received blood transfusion than did those from the restrictive strategy group (77% vs. 43%, *p <* 0.001). Most patients underwent coronary bypass artery grafting (CABG). Patients who were exposed to blood transfusion had lower initial hematocrit and hemoglobin levels compared to patients who did not receive RBC transfusions.

From 502 patients, 236 (47%) patients received transfusion at the operative room, 111 (22%) received at the first postoperative day (1PO), 74 (15%) at the second postoperative day (2PO) and 59 (12%) at the third postoperative day (3PO) (Additional file 2).

**Figure 1:**
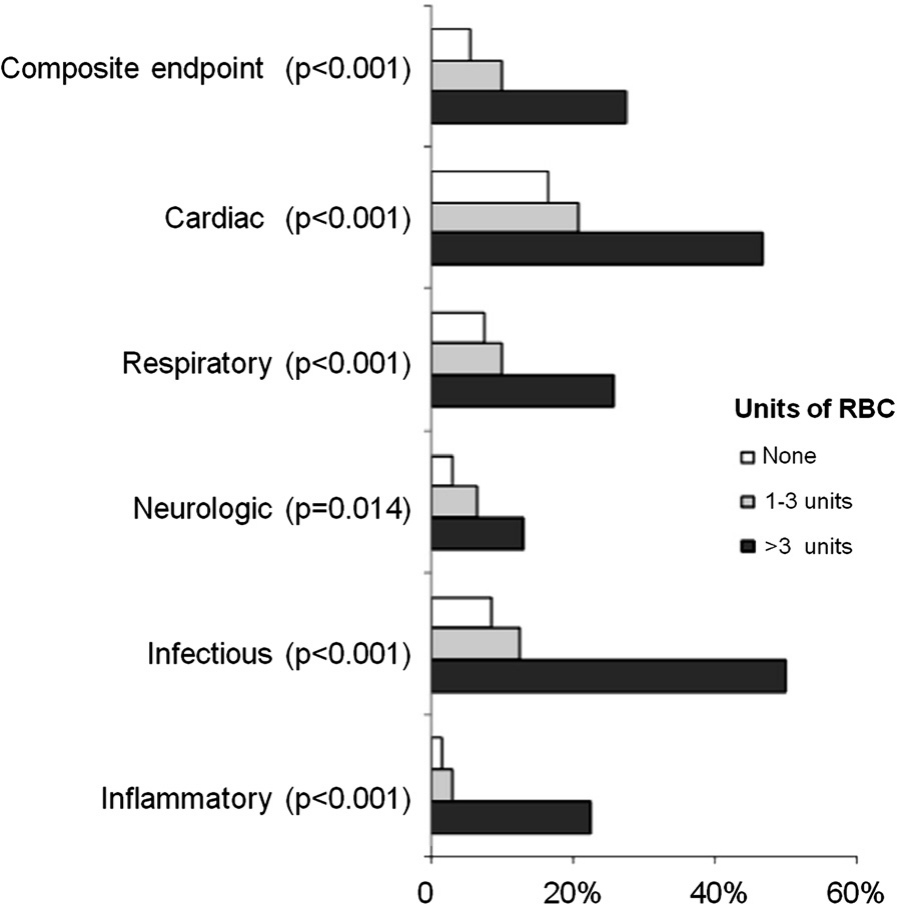
**Complications according to category of red blood cell transfusion.** RBC: red blood cell.

Analyzing postoperative clinical complications, patients who received RBC transfusion were more likely to present major events related to the composite endpoint (30-day all-cause mortality and cardiogenic shock, acute respiratory distress syndrome, or acute kidney injury requiring dialysis or hemofiltration, p < 0.001), in addition to cardiac (p < 0.001), respiratory (p < 0.001), neurologic (p = 0.014), infectious (p < 0.001), and inflammatory complications (p < 0.001) (Figure 1). We also found that patients from the high transfusion group presented a higher incidence of postoperative complications.

### Length of stay analysis

**Figure 2:**
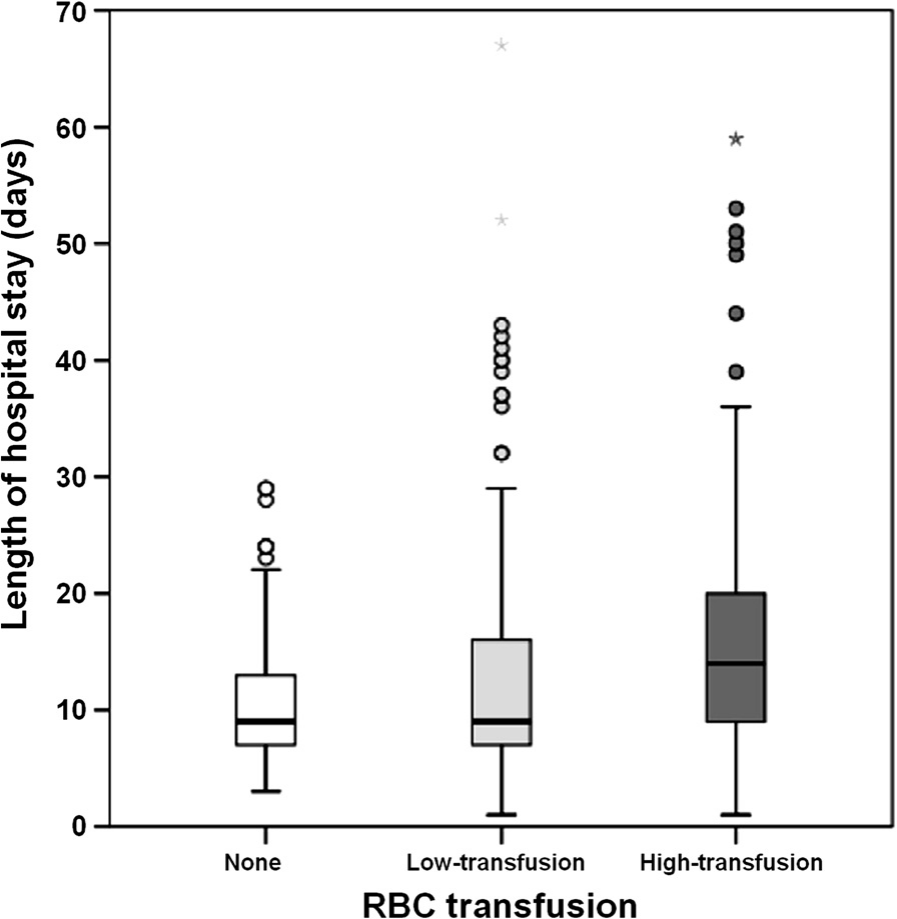
**Box plots of hospital length of stay (LOS), according to category of red blood cell transfusion.** RBC: red blood cell. The boxes indicate the first quartile, the median, and the third quartile. Open circles indicate minor outliers (observation 1.5 × interquartile range [IQR] outside the central box); asterisks indicate major outliers (observation 3.0 × IQR outside the central box).

The median hospital length of stay was 9 days (range, 1 to 67 days). Patients exposed to any RBC transfusion had longer median length of stay than patients in the non-transfusion group: 15 days [95% CI, 12–18] in high transfusion requirement group (> 3 units) vs. 10 days [95% CI, 9–11] in low transfusion requirement group (1–3 units) vs. 9 days [95% CI, 8–10] in non-transfusion group, *p <* 0*.*001) (Figure 2).

Many covariates had statistically significant associations with hospital LOS. These include: female gender, older age, higher EuroSCORE, redo surgery, renal disease, lower initial hemoglobin and hematocrit, lower left ventricular ejection fraction, renal disease, valvular surgery or a combined procedure, longer cardiopulmonary bypass, lower initial hemoglobin and hematocrit, and exposure to RBC transfusion (Additional file 3 and Additional file 4).

All variables significantly associated with increased hospital length of stay were analyzed in a multivariate Cox proportional hazards model. In the multivariate model, the following factors were predictive of a prolonged hospital length of stay: age higher than 65 years (hazard ratio [HR], 1.36 [95% CI, 1.09-1.7]; *p* = 0.006), EuroSCORE higher than 3–5 (HR 1.46 [1.13-1.88]; *p* = 0.003, EuroSCORE higher than 5 (HR, 1.71 [95% CI, 1.27–2.3]; *p* < 0.001), valvular surgery (HR, 1.60 [95% CI, 1.28–1.99]; *p* < 0.001), combined procedure (HR, 1.73 [95% CI, 1.12–2.67]; *p* = 0.013), LVEF lower than 40% (HR, 1.63 [95% CI, 1.19-2.24]; *p* = 0.002), LVEF 40–59% (HR, 1.32 [95% CI, 1.07–1.64]; *p* = 0.001), and RBC transfusion of > 3 units (HR, 2 [95% CI, 1.44–2.79]; *p* < 0.001) (Table 2).

**Table 2:** Multivariate cox proportional hazard model for length of hospital stay

**Variable**	**Hazard ratio**	***p***
	**(95% CI)**	
Age (years)		
<65	Reference	
≥65	1.36 (1.09 – 1.7)	0.006
EuroSCORE		
<3	Reference	
3–5	1.46 (1.13 – 1.88)	0.003
>5	1.71 (1.27 – 2.3)	<0.001
Procedure		
CABG	Reference	
Valve	1.6 (1.28 – 1.99)	<0.001
CABG + valve	1.73 (1.12 – 2.67)	0.013
Left ventricular ejection fraction (%)		
<40	1.63 (1.19 – 2.24)	0.002
40–59	1.32 (1.07 – 1.64)	0.010
≥60	Reference	
Units of RBC transfused		
None	Reference	
Low-transfusion (1–3 units)	1.16 (0.94 – 1.42)	0.164
High-transfusion (>3 units)	2.00 (1.44 – 2.79)	<0.001

Abbreviations: *CI*, confidence interval; *CABG*, coronary artery bypass graft surgery; *min*, minutes; *RBC*; red blood cells.

**Figure 3:**
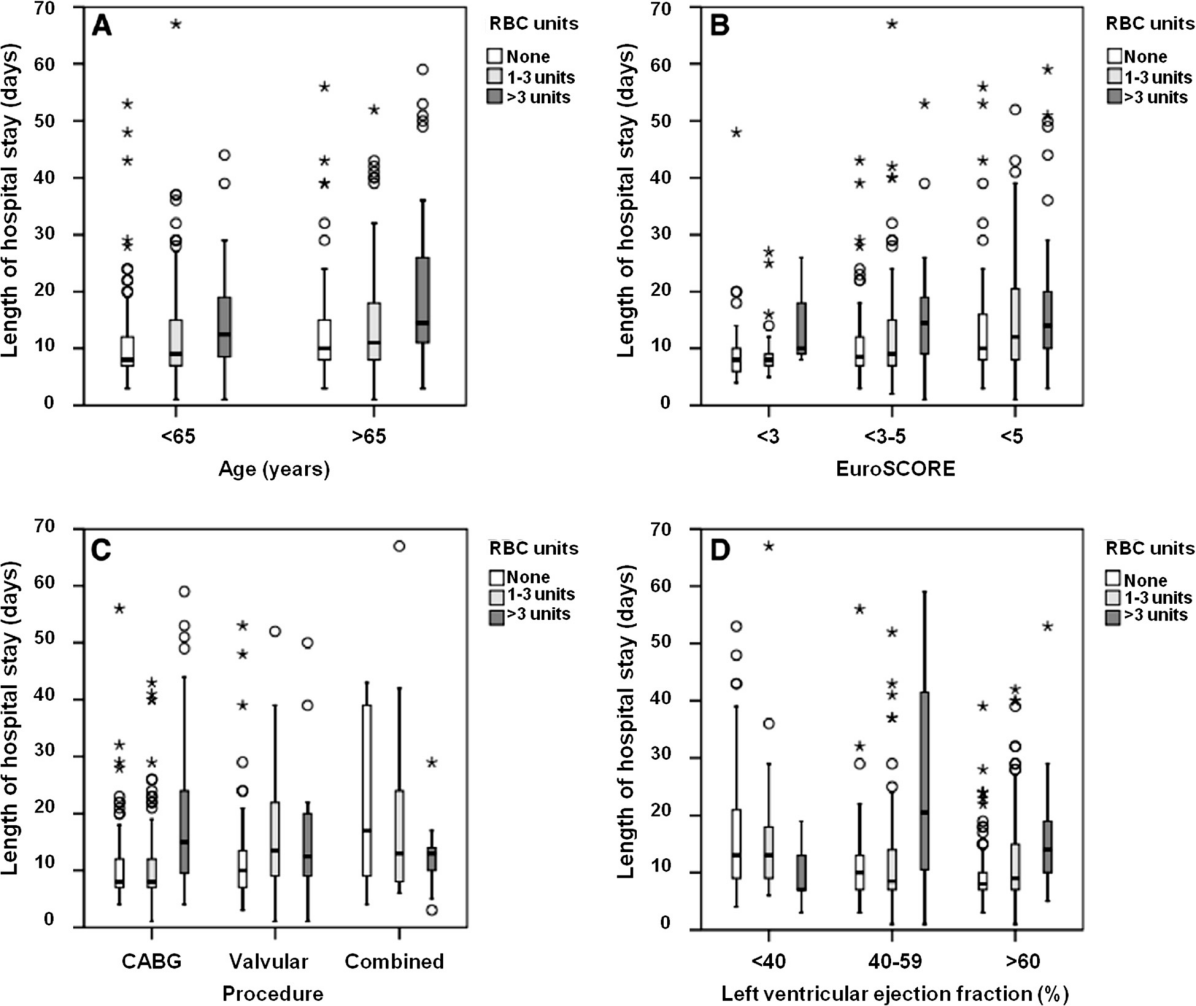
**Box plot indicating the prolonged hospital length of stay according to category of red blood cell transfusion for each of the risk factors: (A) age, (B) EuroSCORE, (C) procedure and (D) left ventricular ejection fraction.** RBC: red blood cell; EuroSCORE: European System for Cardiac Operative Risk Evaluation.

In an adjusted model for age, EuroSCORE, type of surgical procedure and left ventricular ejection fraction, the exposure to RBC transfusion was associated with a prolonged hospital length of stay (Figure 3).

## Discussion

In this study of 502 patients undergoing elective cardiac surgery, we found that 60% received RBC within the first 72 postoperative hours. Patients requiring RBC transfusion were older, most of them were female, underwent redo and complex surgeries, had higher EuroSCORE, presented with lower levels of hemoglobin and hematocrit and had a higher incidence of renal disease. In a multivariable model adjusted for univariate associations, RBC transfusion was a predictor of longer hospital length of stay, and the strongest association was found in the high transfusion group. Independent of the strategy of transfusion, patients who received RBC transfusion in the first 72 postoperative hours had a higher incidence of complications, including renal failure, cardiogenic shock, acute respiratory distress syndrome, infectious, neurologic and inflammatory complications and longer hospital length of stay.

Significant variability in transfusion practice exists in cardiac surgery. A recent analysis [5] of blood practices across 798 US hospitals drew attention to this problem. In hospitals performing at least 100 on-pump CABG operations (82 446 cases), rates of blood transfusion ranged from 7.8% to 92.8% for RBCs, 0% to 97.5% for fresh-frozen plasma, and 0.4% to 90.4% for platelets. Multivariable analysis revealed that after adjustment for patient-level risk factors, hospital transfusion rates varied by geographic location (P = .007), academic status (P = .03), and hospital volume (P < .001).

We found patients who were female, older, underwent redo or complex surgeries, as well as those who had higher EuroSCORE, renal disease, or previous anemia, were more likely to receive RBC transfusion. This finding emphasizes the need to maximize efforts to improve perioperative care in these subgroups of patients in order to avert RBC transfusion adverse events, including increased hospital length of stay.

Rates of transfusion persist high after cardiac surgery despite studies showing that RBC transfusion does not result in better outcomes and even increases rates of complications after cardiac surgery [6]. Guidelines from the Society of Thoracic Surgeons and Society of Cardiovascular Anesthesiologists emphasize the lack of evidence on transfusion triggers after cardiac surgery [7]. Most transfusion indications occur in the first 72 hours after surgery, starting in the operating room, where usually the transfusion indication is due to hemodilution and based on triggers [2].

The rationale for implementing a restrictive transfusion strategy is based on analysis of studies reporting a lack of benefit and, at the same time, substantially increased costs and adverse effects associated with RBC transfusion. These adverse effects include acute hemolytic and nonhemolytic reactions, transmission of viral and bacterial diseases, transfusion-related acute lung injury, and transfusion-associated circulatory overload [8]. Immunosuppression has also been associated with transfusion and may explain the higher risk of infection and recurrence of neoplastic diseases observed in transfused patients [9].

In a retrospective analysis of 11, 963 patients who underwent isolated CABG surgery, Koch et al [10] showed that perioperative RBC transfusion was associated with a dose-dependent increased risk of postoperative cardiac complications, serious infection, renal failure, neurologic complications, overall morbidity, prolonged ventilator support, and in-hospital mortality. In a similar retrospective study, Murphy et al [11] showed that RBC transfusion was strongly associated with infection and with postoperative ischemic morbidity, hospital stay, increased early and late mortality, and hospital costs.

Recently, the TRACS study prospectively demonstrated the safety of a restrictive strategy of RBC transfusion in patients undergoing cardiac surgery [2]. Also, this study reported that the higher the number of transfused RBC, the higher was the number of clinical complications [2].

In our study, we found that patients exposed to RBC transfusion had more complications when compared to patients not exposed, a similar result described in previous reports [1-4,6,10]. The evaluation of mechanisms related to RBC transfusion that results in morbidity were not included in this study. However, our hypothesis it that these potential effects may underlie the independent association between blood transfusion and prolonged LOS. On univariate comparison, total length of stay was approximately 6 days longer in the population of patients who received more than 3 RBC units of transfusion and 1 day longer in patients receiving until 3 units as compared to non-exposed group. Multivariate analysis demonstrated a significant relationship between early postoperative transfusion (more than 3 units of RBC) and longer hospital stay.

De Cocker et al [12]. performed a retrospective analysis of 1566 patients undergoing cardiac surgery and identified age greater than 75 years, female gender, NYHA functional class greater than II, arrhythmias, mitral regurgitation, need for inotropic support or intra-aortic balloon pump, non-elective procedures and aortic surgery as predictive factors for a prolonged ICU stay [12]. However, in this study, blood transfusion was not evaluated as a potential predictor of increased hospital LOS [12].

Prolonged hospital LOS is being studied because it results in increased costs, and clinical complications as exposure to infectious agents. It constitutes a possible method to estimate complications after cardiac surgery [13]. Several models have been developed to predict prolonged stay after cardiac surgery. In our practice, the EuroSCORE is widely applied to predict both mortality and prolonged hospital stay [14,15]. Our findings incorporate RBC transfusion higher than 3 units after cardiac surgery as a strong predictor of prolonged LOS. Also, age higher than 65 years, valvular surgery or a combined procedure, left ventricular ejection fraction lower than 60% were also predictors of increased hospital length of stay, as have been previously reported [15].

Limitations of our study include that it was performed in a single referral center for cardiac surgery, which could compromise the generalizability of our findings. Also, the associations we found between RBC transfusion and longer LOS do not explain causality due to the fact of RBC transfusion may only be a surrogate marker of perioperative complications or higher morbidity. In fact, older and sicker patients with more debilitating conditions were stratified into the transfusion group and it may lead to a misinterpretation. So, it is possible that other variables with impact LOS and not included in the results, may not have been present in the multivariable model. Moreover, the present study is a retrospective analysis from a previous randomized clinical trial with a dictated trigger. Retrospective studies are best done in a large cohort of consecutive unselected patients which could limit our interpretation.

## Conclusion

The increased length of hospital stay related to the number of transfused RBC units supports a restrictive therapy in cardiac surgery. In addition, clinicians caring for patients after cardiac surgery should administer only 1 RBC unit at a time, because this may result in less exposure to risks but similar benefits.

Importantly, although the interpretation of our data must take account the restrictions of the substudy analysis, our results brings additional information regarding an independent association of early RBC transfusion with longer hospital stay.

## Competing interests

The authors declare that they have no competing interests.

## Authors’ contributions

Study concept and design: LAH, FRBGG, JLV, JOCA. Acquisition of data: LAH, FRBGG, REN, CMPDCS, EPMA. Analysis and interpretation of data: LAH, FRBGG, JTF, EAO, JPA, JOCA. Drafting of the manuscript: LAH, EAO, JPA. Critical revision of the manuscript for important intellectual content: FRBGG, CMPDCS, JTF, JOCA. Statistical analysis: JTF. All authors read and approved the final manuscript.

## Supplementary Material

Additional file 1 **Flowchart of screened, excluded and included patients.** TRACS: Transfusion Requirements after Cardiac Surgery; RBC: red blood cells. *Ten patients were excluded after consent was obtained because of a change in surgical plan (i.e., surgery was performed without cardiopulmonary bypass). Click here for file

Additional file 2 **Distribution of the percentage of transfusions according to postoperative day.** Intraop: intraoperatory period, PO: postoperative period. Click here for file

Additional file 3 Preoperative Characteristics Associated with Increased Hospital Length of Stay. Click here for file

Additional file 4 Perioperative Characteristics Associated with Increased Hospital Length of Stay. Click here for file
