# Supplementary material for: Blood transfusion in cardiac surgery is a risk factor for increased hospital length of stay in adult patients
Source: J Cardiothorac Surg. 2013 Mar 26;8:54. doi: 10.1186/1749-8090-8-54 (PMC3639844; doi:10.1186/1749-8090-8-54)
Supplement: Additional file 1 — Flowchart of screened, excluded and included patients. TRACS: Transfusion Requirements after Cardiac Surgery; RBC: red blood cells. *Ten patients were excluded after consent was obtained because of a change in surgical plan (i.e., surgery was performed without cardiopulmonary bypass). [file 1749-8090-8-54-S1.docx]

**E-FIGURES**

**Supplementary Figure 1. Flowchart of screened, excluded and included patients.**


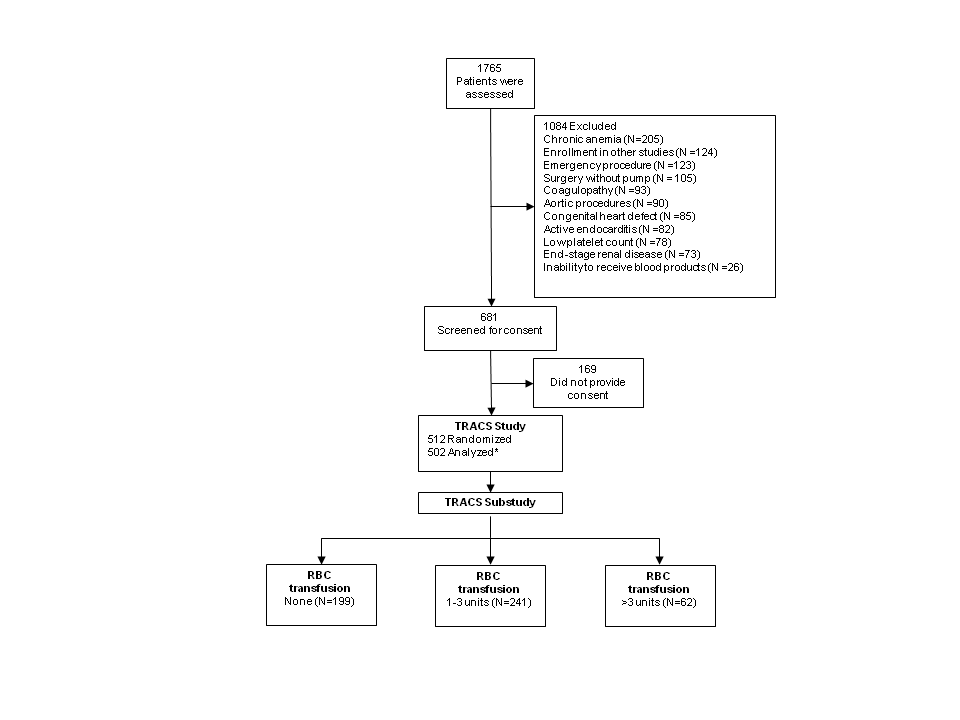


TRACS: Transfusion Requirements after Cardiac Surgery; RBC: red blood cells.

*Ten patients were excluded after consent was obtained because of a change in surgical plan (i.e., surgery was performed without cardiopulmonary bypass).
