# Supplementary material for: Blood transfusion in cardiac surgery is a risk factor for increased hospital length of stay in adult patients
Source: J Cardiothorac Surg. 2013 Mar 26;8:54. doi: 10.1186/1749-8090-8-54 (PMC3639844; doi:10.1186/1749-8090-8-54)
Supplement: Additional file 2 — Distribution of the percentage of transfusions according to postoperative day. Intraop: intraoperatory period, PO: postoperative period. [file 1749-8090-8-54-S2.docx]

**E-FIGURES**

**Supplementary Figure 2E. Distribution of the percentage of transfusions according to postoperative day.**


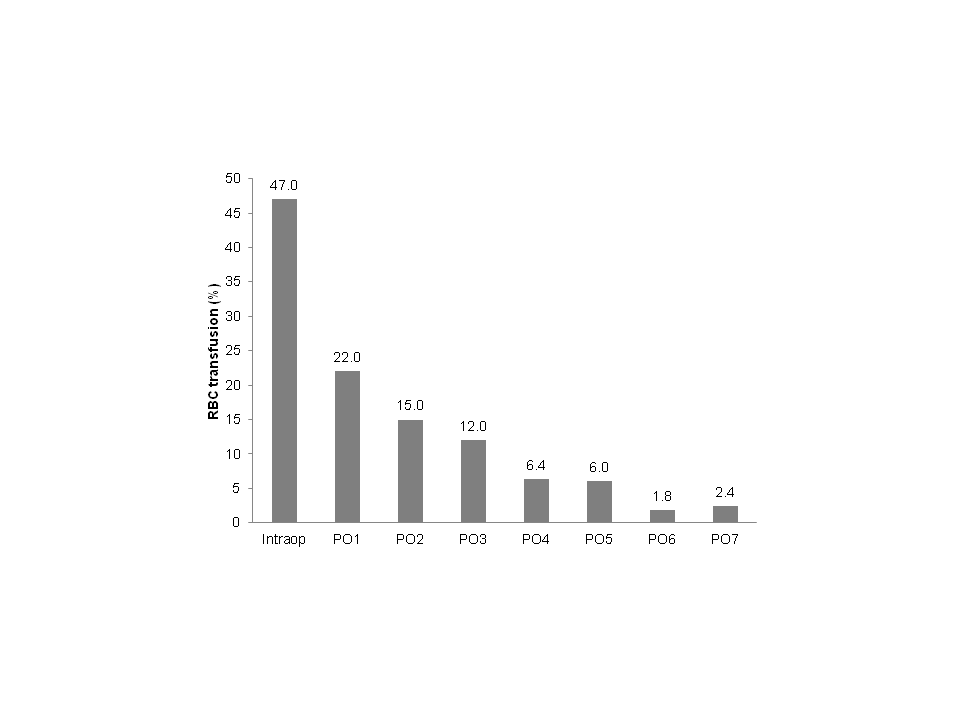


Intraop: intraoperatory period, PO: postoperative period.
