# Supplementary material for: Blood transfusion in cardiac surgery is a risk factor for increased hospital length of stay in adult patients
Source: J Cardiothorac Surg. 2013 Mar 26;8:54. doi: 10.1186/1749-8090-8-54 (PMC3639844; doi:10.1186/1749-8090-8-54)
Supplement: Additional file 3 — Preoperative Characteristics Associated with Increased Hospital Length of Stay. [file 1749-8090-8-54-S3.docx]

**E-TABLES**

**Supplementary Table 1E. Preoperative Characteristics Associated with Increased Hospital Length of Stay**

| Variable | LOS (days) |  | Univariate | |  |
| --- | --- | --- | --- | --- | --- |
|  | Median (95% CI) |  | Hazard ratio (95% CI) | *p* |  |
| Gender |  |  |  |  |  |
| Female | 12 (10.5 - 13.5) |  | 1.41 (1.17 - 1.71) | <0.001 |  |
| Male | 9 (8.4 - 9.6) |  | Reference |  |  |
| Age (years) |  |  |  |  |  |
| <65 | 9 (8.5 - 9.5) |  | Reference |  |  |
| ≥65 | 12 (10.5 - 13.5) |  | 1.54 (1.27 - 1.86) | <0.001 |  |
| EuroSCORE |  |  |  |  |  |
| <3 | 8 (7.4 - 8.6) |  | Reference |  |  |
| 3-5 | 9 (8.2 - 9.8) |  | 1.69 (1.33 - 2.15) | <0.001 |  |
| >5 | 13 (11.6 - 14.4) |  | 2.6 (2 - 3.37) | <0.001 |  |
| Redo surgery |  |  |  |  |  |
| No | 9 (8.4 - 9.6) |  | Reference |  |  |
| Yes | 13 (10.4 - 15.6) |  | 1.34 (1.03 - 1.75) | 0.031 |  |
| Left ventricular ejection fraction (%) | |  |  |  |  |
| <40 | 14 (11.9 - 16.1) |  | 1.78 (1.33 - 2.39) | <0.001 |  |
| 40-59 | 10 (8.6 - 11.4) |  | 1.29 (1.05 - 1.58) | 0.016 |  |
| ≥60 | 9 (8.3 - 9.7) |  | Reference |  |  |
| Previous myocardial infarction |  |  |  |  |  |
| No | 10 (9.2 - 10.8) |  | Reference |  |  |
| Yes | 9 (8.1 - 9.9) |  | 1.05 (0.87 - 1.27) | 0.620 |  |
| Hypertension |  |  |  |  |  |
| No | 9 (7.9 - 10.1) |  | Reference |  |  |
| Yes | 10 (9.3 - 10.7) |  | 1.08 (0.87 - 1.35) | 0.475 |  |
| Diabetes |  |  |  |  |  |
| No | 10 (9.3 - 10.7) |  | Reference |  |  |
| Yes | 10 (8.9 - 11.1) |  | 1.19 (0.98 - 1.45) | 0.077 |  |
| Renal disease |  |  |  |  |  |
| No | 9 (8.4 - 9.6) |  | Reference |  |  |
| Yes | 12 (9.2 - 14.8) |  | 1.42 (1.03 - 1.96) | 0.030 |  |
| Unstable Angina |  |  |  |  |  |
| No | 10 (9.1 - 10.9) |  | Reference |  |  |
| Yes | 9 (8.2 - 9.8) |  | 1.09 (0.89 - 1.32) | 0.418 |  |

Abbreviations: CI, confidence interval; EuroSCORE, European System for Cardiac Operative Risk Evaluation.
