# Supplementary material for: Blood transfusion in cardiac surgery is a risk factor for increased hospital length of stay in adult patients
Source: J Cardiothorac Surg. 2013 Mar 26;8:54. doi: 10.1186/1749-8090-8-54 (PMC3639844; doi:10.1186/1749-8090-8-54)
Supplement: Additional file 4 — Perioperative Characteristics Associated with Increased Hospital Length of Stay. [file 1749-8090-8-54-S4.docx]

**E-TABLES**

**Supplementary Table 2E. Perioperative Characteristics Associated with Increased Hospital Length of Stay**

| Variable | LOS (days) |  | Hazard ratio | |  |
| --- | --- | --- | --- | --- | --- |
|  | Median (95% CI) |  | (95% CI) | *p* |  |
| Red blood cell transfusion strategy | |  |  |  |  |
| Liberal | 10 (9.3 - 10.7) |  | Reference |  |  |
| Restrictive | 10 (9 - 11) |  | 1.03 (0.86 - 1.24) | 0.717 |  |
| Procedure |  |  |  |  |  |
| CABG | 9 (8.5 - 9.5) |  | Reference |  |  |
| Valve | 13 (11.5 - 14.5) |  | 1.45 (1.19 - 1.78) | <0.001 |  |
| CABG + valve | 14 (12.6 - 15.4) |  | 2.05 (1.39 - 3.03) | <0.001 |  |
| Length of cardiopulmonary bypass (min) |  |  |  |  |  |
| <100 | 10 (9.3 - 10.7) |  | Reference |  |  |
| >100 | 10 (9.1 - 10.9) |  | 1.22 (1.01 - 1.49) | 0.040 |  |
| Initial hemoglobin (g/dL) |  |  |  |  |  |
| <13 | 11 (9.7 - 12.3) |  | 1.29 (1.07 - 1.55) | 0.007 |  |
| ≥13 | 9 (8.4 - 9.6) |  | Reference |  |  |
| Initial hematocrit (%) |  |  |  |  |  |
| <39 | 12 (10.6 - 13.4) |  | 1.38 (1.15 - 1.66) | <0.001 |  |
| ≥39 | 9 (8.5 - 9.5) |  | Reference |  |  |
| Initial lactate (mmol/L) |  |  |  |  |  |
| <2 | 10 (9.4 - 10.6) |  | Reference |  |  |
| ≥2 | 10 (8.7 - 11.3) |  | 1.01 (0.81 - 1.24) | 0.962 |  |
| Initial SvO_2_ (%) |  |  |  |  |  |
| <65 | 12 (10.1 - 13.9) |  | 1.17 (0.89 - 1.54) | 0.855 |  |
| ≥65 | 10 (9.3 - 10.7) |  | Reference |  |  |
| Units of RBC Transfused |  |  |  |  |  |
| None | 9 (8.4 - 9.6) |  | Reference |  |  |
| Low-transfusion (1-3 units) | 10 (9 - 11) |  | 1.27 (1.05 - 1.54) | 0.015 |  |
| High-transfusion (>3 units) | 15 (11.9 - 18.1) |  | 2.12 (1.55 - 2.91) | <0.001 |  |

Abbreviations: CI, confidence interval; CABG, coronary artery bypass graft surgery; SvO_2_, central venous oxygen saturation; min, minutes; Initial = samples collected after the induction of anesthesia.
